# Design and Rationale of the National Tunisian Registry of Atrial Fibrillation: Protocol for a Prospective, Multicenter Trial

**DOI:** 10.2196/resprot.8523

**Published:** 2018-10-15

**Authors:** Afef Ben Halima, Sana Ouali, Mohamed Sami Mourali, Sonia Chabrak, Rafik Chettaoui, Manel Ben Halima, Abdeddayem Haggui, Noureddine Larbi, Salma Krichène, Sonia Marrakchi, Slim Kacem, Rim Chrigui, Mohamed Fahmi Abbes, Hédi Baccar, Nadia Baraket, Najeh Ben Halima, Ali Ben Khalfallah, Mohamed Ben Mbarek, Soraya Ben Youssef, Essia Boughzala, Mohamed Rachid Boujnah, Habiba Drissa, Habib Gamra, Ali Gasmi, Habib Haouala, Youssef Harrath, Ines Issa, Gouider Jeridi, Salem Kachboura, Samir Kammoun, Sondes Kraiem, Faouzi Maatouk, Sami Milouchi, Wided Nasraoui, Ali Neji, Khaled Sayahi, Wissem Sdiri, Wajih Smati, Samir Tlili, Leila Abid, Salem Abdesselem, Lilia Zakhama, Abdallah Mahdhaoui, Helmi Kammoun, Skander Ben Omrane, Faouzi Addad

**Affiliations:** 1 Tunisian Society of Cardiology and Cardiac Surgery Tunis Tunisia; 2 La Rabta Hospital Tunis Tunisia; 3 National Tunisian Registry of Atrial Fibrillation Steering Committee Tunisian Society of Cardiology and Cardiac Surgery Tunis Tunisia; 4 Principal Military Hospital Tunis Tunisia; 5 Abderrahman Mami Hospital Ariana Tunisia; 6 Zaghouan Hospital Zaghouan Tunisia; 7 Charles Nicolle Hospital Tunis Tunisia; 8 Mohamed Taher Al Maamouri Hospital Nabeul Tunisia; 9 Ibn El Jazzar Hospital Kairouan Tunisia; 10 Menzel Bourguiba Hospital Menzel Bourguiba Tunisia; 11 Kebili Hospital Kebili Tunisia; 12 Hospital of the Internal Security Forces La Marsa Tunisia; 13 Sahloul Hospital Sousse Tunisia; 14 Mongi Slim Hospital La Marsa Tunisia; 15 Fattouma Bourguiba Hospital Monastir Tunisia; 16 Mohamed Ben Sassi Hospital Gabes Tunisia; 17 Siliana Hospital Siliana Tunisia; 18 Grombalia Hospital Nabeul Tunisia; 19 Farhat Hached Hospital Sousse Tunisia; 20 Hédi Chaker Hospital Sfax Tunisia; 21 Habib Thameur Hospital Tunis Tunisia; 22 Habib Bourguiba Hospital Medenine Tunisia; 23 Kasserine Hospital Kasserine Tunisia; 24 Ben Guerdane Hospital Ben Guerdane Tunisia; 25 Kef Hospital Kef Tunisia; 26 Bougatfa Hospital Bizerte Tunisia; 27 Houssine Bouzaiene Hospital Gafsa Tunisia; 28 Hédi Jaballah Hospital Tozeur Tunisia

**Keywords:** atrial fibrillation, registry, North African, NATURE-AF

## Abstract

**Background:**

Atrial fibrillation (AF) is an important health problem in Tunisia. A significant change in the epidemiological pattern of heart disease has been seen in the last 3 decades; however, no large prospective multicenter trial reflecting national data has been published so far. Robust data on the contemporary epidemiological profile and management of AF patients in Tunisia are limited.

**Objective:**

The aim of this study is to analyze, follow, and evaluate patients with AF in a large multicenter nationwide trial.

**Methods:**

A total of 1800 consecutive patients with AF by electrocardiogram, reflecting all populations of all geographical regions of Tunisia, will be included in the study, with the objective of describing the epidemiological pattern of AF. Patients will be officially enrolled in the National Tunisian Registry of Atrial Fibrillation (NATURE-AF) only if an electrocardiogram diagnosis (12-lead, 24-hour Holter, or other electrocardiographic documentation) confirming AF is made. The qualifying episode of AF should have occurred within the last year, and patients do not need to be in AF at the time of enrollment. Patients will be followed for 1 year. Incidence of stroke or transient ischemic attack, thromboembolic events, and cardiovascular death will be recorded as the primary end point, and hemorrhagic accidents, measurement of international normalized ratio, and time in therapeutic range will be recorded as secondary end points.

**Results:**

Results will be available at the end of the study; the demographic profile and general risk profile of Tunisian AF patients, frequency of anticoagulation, frequency of effective treatment, and risks of thromboembolism and bleeding will be evaluated according to the current guidelines. Major adverse events will be determined. NATURE-AF will be the largest registry for North African AF patients.

**Conclusions:**

This study would add data and provide a valuable opportunity for real-world clinical epidemiology in North African AF patients with insights into the uptake of contemporary AF management in this developing region.

**Trial Registration:**

ClinicalTrials.gov NCT03085576; https://clinicaltrials.gov/ct2/show/NCT03085576 (Archived by WebCite at http://www.webcitation.org/6zN2DN2QX)

**Registered Report Identifier:**

RR1-10.2196/8523

## Introduction

### Background

Atrial fibrillation (AF) is the most common sustained cardiac rhythm disorder, and recent projections in Europe estimate that from 2010 to 2060, the number of adults aged 55 years and older with AF in the European Union will more than double [1]. With the aging population and associated prevalence of other cardiovascular diseases, the burden of AF is projected to increase. It is estimated that by 2050, the prevalence of AF in Africa will be greater than in any other region of the world [2]. Given the increasing prevalence and AF’s association with significant morbidities and mortality, this increase would have major public health implications.

In the last decades, a significant change in the epidemiologic and etiologic patterns of cardiovascular diseases has been seen in North Africa with a decrease in rheumatic heart disease and increase in hypertensive and ischemic heart disease [2,3]. The World Health Organization reported trends in the incidence and prevalence of acute rheumatic fever and rheumatic heart disease for each continent based on literature from 100 countries around the world between 1970 and 2009 [4,5]. However, data from Africa are scarce and do not capture the entire time frame. As for all heart diseases, there are insufficient contemporary population-based data describing the epidemiologic pattern of AF in North Africa and especially in Tunisia. In 2003, valvular AF secondary to rheumatic heart disease was the most common etiologic form of AF [6].

Numerous registries and surveys have been described in different European, Asian, and American countries—Euro Observational Research Programme–Atrial Fibrillation pilot general registry [7], Japanese Rhythm Registry [8], Global Anticoagulant Registry in the Field [9], Global Registry on Long-Term Oral Antithrombotic Treatment in Patients with Atrial Fibrillation [10], the nationwide US Practice Innovation and Clinical Excellence Registry [11], Outcomes Registry for Better Informed Treatment of Atrial Fibrillation [12], and Chinese Atrial Fibrillation Registry study [13]. However, few data on the demographic characteristics, outcome of AF patients, and quality of anticoagulation control achieved in AF patients receiving routine medical care are available in North Africa and especially in Tunisia.

Demographic and prognostic AF data from other ethnic groups would not be generalizable to our population. Thus, a register or a survey dealing with the demographic and prognostic characteristics of AF in Tunisia is essential, making it possible to identify its specific characteristics inherent in part to ethnic particularities but especially to particularities of the local health system.

### Registry Objectives

The National Tunisian Registry of Atrial Fibrillation (NATURE-AF) is a prospective observational accumulation of data used in the investigation of the optimal intensity of anticoagulation in Tunisian AF patients and present status of anticoagulation treatment in Tunisia.

The primary end point of NATURE-AF is to describe the incidence of stroke or transient ischemic attack (TIA), thromboembolic event, and cardiovascular death every 3 months up to 1 year.

The secondary end points are as follows:

- Hemorrhagic accidents, every 3 months up to 1 year
- International normalized ratio (INR) every month for 1 year
- Mean time in therapeutic range (TTR) obtained in patients who receive anticoagulant therapy

## Methods

### Study Design and Patient Enrollment

NATURE-AF is a prospective, observational registry with a 1-year follow-up period. The enrollment occured all over Tunisia between March 1, 2017, and May 31, 2017. The registry population comprised consecutive in- and outpatients with AF presenting to cardiologists. Consecutive patients were screened for eligibility at the time of their presentation to a cardiologist (hospital or medical center). All patients provided written informed consent. Patients were officially enrolled in NATURE-AF only if they were aged 20 years and older and had had at least 1episode of AF recorded on a standard 12-lead electrocardiogram or on 24-hour Holter monitor. The qualifying episode of AF should have occurred within the last year and could be valvular or nonvalvular AF. Valvular AF is AF in patients with mitral stenosis or prosthetic heart valves. Patients did not need to be in AF at the time of enrollment. All patients admitted for catheter ablation, initiation of drug therapy, or cardioversion (electrical or pharmacological) were eligible to be included.

Exclusion criteria were AF due to reversible causes (eg, thyroid disease and pulmonary embolism) including postoperative AF (≤3 months), life expectancy less than 12 months, acute coronary syndrome, isolated atrial flutter, mental disorders, and ongoing anticoagulation for reasons other than AF.

### Sample Size and Data Collection

A minimum of 10 consecutive patients per cardiologist were enrolled with a target of 1800 patients for NATURE-AF. A total of 186 cardiologists agreed to participate.

While it was anticipated that most investigators would be hospital-based cardiologists, recruitment by office-based cardiologists was allowed if follow-up of patients was deemed feasible.

The plan was to have 1 baseline visit and 1 visit every 3 months over a 1-year period. Enrollment into the registry started March 1, 2017, with an inclusion period estimated up to 3 months. All patients were followed for 12 months. During this period, all participants revisited their cardiologists at the usual intervals (3 months), and patients taking oral anticoagulant therapy consult (or visited) at least once every month for INR to be measured.

The data collected were managed by the Clinical Suite platform (Dacima Software), which complies with international standards including US Food and Drug Administration 21 Code of Federal Regulations Part 11, US Health Insurance Portability and Accountability Act, International Conference on Harmonisation, and Medical Dictionary for Regulatory Activities. The Clinical Suite platform allowed us to track the data entered, check for inconsistencies and missing data, and schedule monitoring visits. A steering committee was set up to monitor patient inclusions, verify data sources, perform the audit trail, and prepare the statistical analysis plan for the study. Data were collected every 3 months regardless of patient clinic follow-up. All incident events and therapeutic changes were entered at each collection interval.

Baseline data included patient demographics, medical history, cardiovascular history, details of AF history and therapies, vital signs, laboratory measurements, electrocardiographic data, cardiac imaging parameters, details of medical management, and any contraindications to anticoagulation. At follow-up, major incident events and procedures, subsequent vital signs, laboratory studies, imaging parameters, and medication changes were recorded, and the daily acenocoumarol (Sintrom) dose and INR value were noted for all patients taking acenocoumarol. In-depth data regarding antithrombotic therapies, dosing, discontinuations, and reasons for discontinuations were included in follow-up medication data.

### Timeline

**Figure 1:**
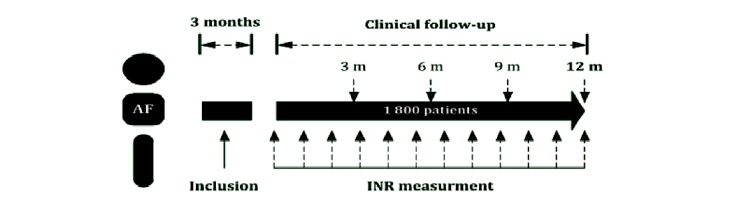
Study protocol AF: atrial fibrillation; INR: international normalized ratio.

Patient enrollment and data collection began in March 2017 and continued until the end of May 2017. Follow-up continued until all patients had 1-year data. Figure 1 describes the study protocol.

### Outcomes

During follow-up, the end points of this observational study were symptomatic stroke including TIA, systemic thromboembolism, myocardial infarction, incident heart failure, cause-specific hospitalization, major bleeding, and all causes of death.

Major bleeding is defined by the International Society of Thrombosis and Hemostasis criteria; this includes bleeding events meeting at least one of the following criteria [14]:

- Decrease in hemoglobin ≥2 g/dL
- Transfusion of ≥2 units of packed red blood cells or whole blood
- Any bleeding in a critical site (intracranial, intraspinal, intraocular, intra-articular, pericardial, retroperitoneal, or intramuscular with compartment syndrome)
- Any fatal bleeding

Additional unique, detailed data on management of bleeding events were collected and included the use of any blood products or transfusions, potential reversal agents, and necessity for invasive management of bleeding events. Any patient in whom a primary end point was encountered was evaluated by computed tomography or magnetic resonance imaging for precise diagnosis and required an INR value on the closest possible day to their revisit day to their cardiologist.

INR will be recorded monthly and TTR will be calculated according to Roosendaal’s algorithm with linear interpolation [15].

### Ethical Considerations

Ethics approval was obtained from the Human Research Ethics Committees at Abderrahmen Mami Hospital in Tunis. Ethics review boards in each participating hospital approved their participation. Informed consent from individual patients was obtained before participation in long-term follow-up. The study was performed according to the ethical principles for medical research involving human subjects specified in the Declaration of Helsinki and International Conference on Harmonisation Good Clinical Practices.

### Statistical Analysis

Continuous variables will be described by mean and standard deviation or as median and interquartile range. Categorical variables will be described by the size and frequency of every modality. Means comparison will be performed by analysis of variance or by nonparametric tests if the hypothesis of normality is rejected. The normality of continuous variables will be verified with the Shapiro-Wilk test. The statistical tests are bilateral with a 95% confidence interval.

A chi-square test will be performed for categorical variables. Yates correction or the Fisher exact test will be used if the conditions of validity for the chi-square test are not met.

A multivariate analysis will be performed with anticoagulant treatment (over or undertreated) as dependant factor. The independent variables will be age, gender, body mass index, type of AF, combined therapy. Univariate logistic regression will be carried out with a 10% output threshold. The final model will be performed with the parameters selected by the backward stepwise method of Wald. The selected variables in the final model will be tested at the 5% threshold. The interaction between selected parameters is tested at the 10% threshold.

The TTR will be calculated by the method first described by Rosendaal et al [8], which uses linear interpolation of INR values in each patient under oral anticoagulant treatment to calculate the percentage of days when the INR is in the therapeutic range (2.0-3.0) for nonvalvular AF.

### Expected Implications

The NATURE-AF is the first large-scale investigation to clarify the contemporary demographic data, management and outcomes of AF patients, and frequency and quality of oral anticoagulation in Tunisian AF patients.

### Oversight and Leadership

The protocol of NATURE-AF was approved by the Tunisian Society of Cardiology and Cardiovascular Surgery. The NATURE-AF study was submitted to ClinicalTrials.gov [NCT03085576].

### Study Sponsorship

NATURE-AF is sponsored by the Tunisian Society of Cardiology and Cardiovascular Surgery.

## Results

About 95 cardiologists included 918 patients in the registry with a 1-year follow-up period. All patients provided written informed consent. Patients were officially enrolled in NATURE-AF only if they were aged 20 years and older and had had at least 1 episode of AF recorded on a standard 12-lead electrocardiogram or on 24-hour Holter monitor.

## Discussion

### Summary

Numerous registries and surveys have been described in different European, Asian, and American countries [7-13,16-19], but few contemporary data on the demographic characteristics, outcome of AF patients, and quality of anticoagulation control achieved in AF patients receiving routine medical care are available in North Africa and especially in Tunisia.

Only 2 published studies have described the epidemiological data on Tunisia [5,15,16]. In 2003, Drissaet al [6] described a multicentric study with 1134 patients presenting with a first episode of AF between January 1985 and December 2000. The average age was 58.6 (SD 15-60) years; 57.8% (656/1134) were male and 42.2% (478/1134) were female. The most common etiology of AF identified was rheumatic carditis (36.1%). AF was idiopathic in 27.7% of cases. Higher morbidity and mortality were demonstrated in AF patients with a 5-year survival of 85%.

Recently, geographic differences have been highlighted by Gamra et al [16,17]. The RealiseAF international cross-sectional survey enrolled 10,523 patients (with at least 1 documented AF episode in the preceding 12 months) from 831 sites; 26 countries from 4 continents participated in the study with Middle East and North Africa participation from Algeria (n=310), Egypt (n=458), Lebanon (n=191), Morocco (n=250), and Tunisia (n=471). AF patients from the Middle East and Africa were significantly younger and more frequently female compared with those originating from the rest of the world. A CHADS_2_ (congestive heart failure, hypertension, age >75 years, diabetes mellitus, stroke) score ≥2 was observed in 64.2% of the patients originating from Europe versus 58.3%, 57.8%, and 43.6% from Latin America, Asia, and the Middle East and Africa, respectively. Among those patients with a CHADS_2_ score ≥2, there were also important geographical differences with respect to the use of antithrombotics: the proportion of patients not receiving any antithrombotic therapy ranged from 11.4% in the Middle East and Africa to 27.6% in Latin America. Conversely, the use of oral anticoagulants was highest in the Middle East and Africa (66.7%) and lowest in Asia (31.7%) [16,17].

Despite the many complexities associated with the use of vitamin K antagonists (VKA), it remains a mainstay of anticoagulation therapy. Acenocoumarol, a derivative of coumarin, is the most popular VKA used in Tunisia and numerous countries around the world. Maintaining therapeutic range in patients treated with VKAs has always been challenging, and the potential consequences of deviating from the therapeutic range are deleterious.

Although not easily achieved, high anticoagulation control, expressed as TTR, has a paramount effect on patient outcomes, reducing stroke events and mortality rates.

This large, contemporary longitudinal study of Tunisian AF patients will provide a unique opportunity to answer many clinical questions. The NATURE-AF study is important in several respects. First, systematic observational and outcomes data can be generated from this registry study, which is especially valuable given that evidence for Tunisian AF patients is limited. Second, treatment of AF is changing dramatically, and AF management needs to be evaluated in real-world studies. Third, the NATURE-AF study provides a good opportunity to compare treatment and response variation among AF populations in Africa for comparison with different countries and evaluate adherence to recent guidelines.

### Conclusions

NATURE-AF will fill a significant gap in the dynamic landscape of AF care and research. It will provide unique and necessary data on the management and outcomes of AF patients treated. This study will yield the largest contemporary longitudinal cohort of patients with AF in Tunisia and would provide a valuable opportunity for real-world clinical epidemiology with insights into the uptake and outcomes of contemporary AF management.

## Abbreviations

AF: atrial fibrillation
CHADS_2_: congestive heart failure, hypertension, age >75 years, diabetes mellitus, stroke
INR: international normalized ratio
NATURE-AF: National Tunisian Registry of Atrial Fibrillation
TIA: transient ischemic attack
TTR: time in therapeutic range
VKA: vitamin K antagonist

## References

[1] Krijthe BP, Kunst A, Benjamin EJ, Lip GYH, Franco OH, Hofman A, Witteman JCM, Stricker BH, Heeringa J (2013). Projections on the number of individuals with atrial fibrillation in the European Union from 2000 to 2060. Eur Heart J.

[2] Stambler BS, Ngunga LM (2015). Atrial fibrillation in Sub-Saharan Africa: epidemiology, unmet needs, and treatment options. Int J Gen Med.

[3] Rahman F, Kwan GF, Benjamin EJ (2014). Global epidemiology of atrial fibrillation. Nat Rev Cardiol.

[4] Seckeler MD, Hoke TR (2011). The worldwide epidemiology of acute rheumatic fever and rheumatic heart disease. Clin Epidemiol.

[5] Rothenbühler M, O'Sullivan CJ, Stortecky S, Stefanini GG, Spitzer E, Estill J, Shrestha NR, Keiser O, J&uuml;ni P, Pilgrim T (2014). Active surveillance for rheumatic heart disease in endemic regions: a systematic review and meta-analysis of prevalence among children and adolescents. Lancet Glob Health.

[6] Drissa H, Essafi N, Mahjoub H, Ben FM, Boujnah MR, Haouala H, Kamoun S, Khalfallah A, Slimane ML, Zaouali RM (2003). Multicenter study of atrial fibrillation. Tunis Med.

[7] Lip GYH, Laroche C, Dan G, Santini M, Kalarus Z, Rasmussen LH, Oliveira MM, Mairesse G, Crijns HJGM, Simantirakis E, Atar D, Kirchhof P, Vardas P, Tavazzi L, Maggioni AP (2014). A prospective survey in European Society of Cardiology member countries of atrial fibrillation management: baseline results of EURObservational Research Programme Atrial Fibrillation (EORP-AF) Pilot General Registry. Europace.

[8] Atarashi H, Inoue H, Okumura K, Yamashita T, Origasa H, J-RHYTHM Registry Investigators (2011). Investigation of optimal anticoagulation strategy for stroke prevention in Japanese patients with atrial fibrillation—the J-RHYTHM Registry study design. J Cardiol.

[9] Kakkar AK, Mueller I, Bassand J, Fitzmaurice DA, Goldhaber SZ, Goto S, Haas S, Hacke W, Lip GYH, Mantovani LG, Verheugt FWA, Jamal W, Misselwitz F, Rushton-Smith S, Turpie AGG (2012). International longitudinal registry of patients with atrial fibrillation at risk of stroke: Global Anticoagulant Registry in the FIELD (GARFIELD). Am Heart J.

[10] Huisman MV, Lip GYH, Diener HC, Dubner SJ, Halperin JL, Ma CS, Rothman KJ, Teutsch C, Zint K, Ackermann D, Clemens A, Bartels DB (2014). Design and rationale of Global Registry on Long-Term Oral Antithrombotic Treatment in Patients with Atrial Fibrillation: a global registry program on long-term oral antithrombotic treatment in patients with atrial fibrillation. Am Heart J.

[11] Hsu JC, Maddox TM, Kennedy KF, Katz DF, Marzec LN, Lubitz SA, Gehi AK, Turakhia MP, Marcus GM (2016). Oral anticoagulant therapy prescription in patients with atrial fibrillation across the spectrum of stroke risk: insights From the NCDR PINNACLE registry. JAMA Cardiol.

[12] Steinberg BA, Blanco RG, Ollis D, Kim S, Holmes DN, Kowey PR, Fonarow GC, Ansell J, Gersh B, Go AS, Hylek E, Mahaffey KW, Thomas L, Chang P, Peterson ED, Piccini JP, ORBIT-AF Steering Committee Investigators (2014). Outcomes Registry for Better Informed Treatment of Atrial Fibrillation II: rationale and design of the ORBIT-AF II registry. Am Heart J.

[13] Du X, Ma C, Wu J, Li S, Ning M, Tang R, Guo X, Long D, Yu R, Sang C, Jiang C, Zhang T, Pan J, Liu X, Dong J, Lip GYH, CAFR Investigators (2016). Rationale and design of the Chinese Atrial Fibrillation Registry study. BMC Cardiovasc Disord.

[14] Schulman S, Kearon C, Subcommittee on Control of Anticoagulation of the Scientific Standardization Committee of the International Society on Thrombosis and Haemostasis (2005). Definition of major bleeding in clinical investigations of antihemostatic medicinal products in non-surgical patients. J Thromb Haemost.

[15] Rosendaal FR, Cannegieter SC, van der Meer FJ, Briët E (1993). A method to determine the optimal intensity of oral anticoagulant therapy. Thromb Haemost.

[16] Gamra H, Murin J, Chiang C, Naditch-Brûlé L, Brette S, Steg PG, RealiseAF investigators (2014). Use of antithrombotics in atrial fibrillation in Africa, Europe, Asia and South America: insights from the International RealiseAF Survey. Arch Cardiovasc Dis.

[17] Chiang C, Naditch-Brûlé L, Brette S, Silva-Cardoso J, Gamra H, Murin J, Zharinov OJ, Steg PG (2016). Atrial fibrillation management strategies in routine clinical practice: insights from the international RealiseAF survey. PLoS One.

[18] Ertaş F, Kaya H, Kaya Z, Bulur S, Küse N, Gül M, Kahya EN, Cağlıyan CE, Köroğlu B, Vatan B, Acar G, Yüksel M, Bilik MZ, Gedik S, Simşek Z, Akıl MA, Yılmaz R, Oylumlu M, Arıbaş A, Yıldız A, Aydın M, Yeter E, Kanadaşı M, Ergene O, Ozhan H, Ulgen MS (2013). Epidemiology of atrial fibrillation in Turkey: preliminary results of the multicenter AFTER study. Turk Kardiyol Dern Ars.

[19] Lara-Vaca S, Cordero-Cabra A, Mart&iacute;nez-Flores E, Iturralde-Torres P (2014). [The Mexican Registry of Atrial Fibrillation (ReMeFa)]. Gac Med Mex.

